# Structure and Dynamics of the Breast Tissue Microbiomes Under Tumor Influences: An Approach With Neutral, Near-Neutral, and Niche-Neutral Hybrid Models

**DOI:** 10.3389/fmicb.2021.614967

**Published:** 2021-07-19

**Authors:** Lianwei Li, Ping Ning, Zhanshan (Sam) Ma

**Affiliations:** ^1^Computational Biology and Medical Ecology Lab, State Key Laboratory of Genetic Resources and Evolution, Kunming Institute of Zoology, Chinese Academy of Sciences, Kunming, China; ^2^Chengdu Women’s and Children’s Central Hospital, School of Medicine, University of Electronic Science and Technology of China, Chengdu, China; ^3^Center for Excellence in Animal Evolution and Genetics, Chinese Academy of Sciences, Kunming, China

**Keywords:** niche-neutral hybrid model, multi-site neutral model, species-level neutrality analysis, tissue microbiomes, breast tumor

## Abstract

The structure and dynamics of breast tissue bacteria can have far-reaching influences on women’s health, particularly on breast tumor development. However, there is little understanding on the ecological processes that shape the structure and dynamics of breast tissue bacteria. Here, we fill the gap by applying three metacommunity models for investigating the community assembly and diversity maintenance, including Sloan near neutral model, Harris et al. multisite neutral and Tang & Zhou niche-neutral hybrid models to reanalyze the 16S-rRNA sequencing datasets of 23 healthy, 12 benign tumor, and 33 malignant tumor tissue samples. First, we found that, at the community/metacommunity levels, the mechanisms of bacteria assembly and diversity maintenance of breast tissue bacteria were moderately influenced by stochastic drifts of bacteria demography (division, death, and dispersal of bacterial cells). At species level, on average, approximately 10 and 5% species were above (positively selected) and below (negatively selected) neutral, respectively. Furthermore, malignant tumor may raise the positively selected species up to 17%. Second, malignant tumor appears to inhibit microbial dispersal as evidenced by lowered migration rates, compared with the migration in normal and benign tumor tissues. These theoretic findings can be inspirational for further investigating the relationships between tissue bacteria and breast tumor progression/development.

## Introduction

With the rapid development of 16S rRNA gene amplicon sequencing, the microbiome of breast milk has become an area of interest for research on the health of infants and mothers. The microbiome in human milk may be the seed source of gut microbiota in infants, with its composition and diversity found to be correlated with body-mass index (BMI), parity, mode of delivery, breastfeeding practices, and infant oral cavities. For example, breastfeeding mode is a key determining factor of milk microbiota composition (Moossavi et al., 2019). Furthermore, microbiomes can influence susceptibility to cancers and the response to therapeutics (Helmink et al., 2019). Many researchers have focused on the relationship between microbial profiles and disease development. Human milk and breast tissue contain microbial communities that are thought to be sterile (Hunt et al., 2011; Urbaniak et al., 2014, 2016; Hieken et al., 2016). Evidence indicates that distinct microbial communities exist among healthy, benign, and malignant breast tissue. Hunt et al. (2011) explored the microbial profiles in human milk based on pyrosequencing of the 16S ribosomal RNA gene and found that the milk microbiome is relatively stable over time within an individual. However, this is not always true. Ma et al. (2015) re-analyzed bacterial interactions using the same datasets as Hunt et al. (2011) and found that dysbiosis of the milk microbiome following a shift in the balance between potential opportunistic pathogens and harmless bacteria is likely responsible for some breast diseases. The gut microbiome can also affect breast cancer due to the estrobolome, i.e., bacterial genes capable of metabolizing estrogens, and thus can affect the emergence of estrogen-driven breast cancers (Goedert et al., 2015; Kwa et al., 2016).

As described previously, breastfeeding and estrogen can impact the breast microbiome profile and diversity. It has been reported that diversity in the milk microbial community is decreased in Hodgkin’s lymphoma cohorts compared with healthy controls, and is positively correlated with milk metabolites that benefit children and negatively correlated with harmful metabolites related to mastitis and breast cancer (Ma et al., 2016). Obviously, the interaction between microbes and breast disease can change the composition and diversity of the microbiome. However, the mechanisms of breast microbiome construction in healthy and diseased groups remain unclear.

To explore the mechanisms of microbiome construction and diversity maintenance, niche theory and neutral theory can be applied to illustrate the role of stochastic and deterministic forces. Hubbell (2001) initially applied the unified neutral theory of biodiversity and biogeography (UNTB) to explain the mechanisms driving the community structure, with many researchers subsequently extending and challenging traditional neutral and niche theories (McGill, 2003; Volkov et al., 2003, 2007; Etienne, 2005, 2007; Hubbell, 2006; Sloan et al., 2006, 2007; Tang and Zhou, 2013; Burns et al., 2016; Li and Ma, 2016; Harris et al., 2017). For example, Sloan et al. (2006, 2007) extended Hubbell’s discrete neutral theory to a continuous version to test large microbiomes. This model can define whether a species is neutral or not beyond the community level and can be applied to identify important microbes in the community. Recently, the Neutral models have been utilized to explore the mechanisms that shape the community structure from human, animal, plants and environments (Burns et al., 2016; Li and Ma, 2016; Chen et al., 2017; Dai et al., 2017; Harris et al., 2017; Li et al., 2018; Ma and Li, 2019; Li L. W. and Ma, 2020; Li W. D. and Ma, 2020; Ma, 2020a, b).

In the current study, we applied Sloan’s neutral community model to define neutral and non-neutral species in healthy (control), benign, and malignant tumor cohorts and to explain the dynamics of neutral species. Compared with the original Hubbell (2001) UNTB model, Sloan et al. (2006; 2006) model is actually a *near neutral* model since it allows for the existence of competitive advantages or disadvantages. Thanks to this advance, all species in a community can be categorized as three types: neutral species, negatively selected (under neutral) and positively selected (above neutral). We take advantage of this feature to detect the potential correlation between tumor development and bacterial species competitiveness. We also used another pair of models, i.e., multi-site neutral (MSN) (Harris et al., 2017) and niche-neutral hybrid models (NNH) (Tang and Zhou, 2013), to evaluate the relative significance of stochastic neutral drifts vs. deterministic niche selection in driving community assembly and shaping the diversity patterns of the breast tissue microbiome. The MSN by Harris et al. (2017) is a major computational advance to Hubbell’s classic UNTB because it allows for simultaneously estimation of the migrations rates among large number of sites (local communities), which was a significant computational challenge until recently. Obviously, this simultaneous estimation of migration parameters is closer to reality. While the MSN model is an orthodox implementation of Hubbell’s UNTB model, and Sloan model is a near neutral model, the NNH model is a mixture (hybrid) of neutral and niche mechanisms. Therefore, the three models we choose to apply in this study span the whole spectrum of the so-termed *niche-neutral continuum*, which postulates that different types of metacommunities are likely fall in different locations of the continuum. One end of the spectrum is occupied by the completely neutral assemblages, and another end is by completely niche-differentiated assemblages.

Therefore, the objective of this article is to examine the “position” of the bacterial communities of breast tissue on the niche-neutral continuum, particularly when tumor development occurs with the breast tissue. The integrated analysis with the three models allows us to present a relatively complete and reliable “picture” of the process (mechanism) underlying the tissue bacteria distribution and dispersal patterns. In perspective, our study, if successful, is likely to offer important insights for investigating the relationship between breast tumor development and breast tissue bacteria (Nejman et al., 2020; Poore et al., 2020).

## Materials and Methods

### Microbiome in Normal, Benign, and Malignant Breast Tissue

Urbaniak et al. (2016) collected samples from women following lumpectomies or mastectomies and from healthy individuals. The breast tissue bacteria datasets consisted of three groups: i.e., healthy (23 samples), benign tumor (12 samples), and malignant tumor tissues (33 samples). Samples were taken from normal tissue adjacent to the tumor, rather than from the tumor tissue itself. The DNA was extracted from breast tissues, and V6 region of 16S rRNA gene was sequenced using Illumina MiSeq platform. A series of procedures for quality control were used to filter raw sequencing data (Urbaniak et al., 2016). We downloaded the sequencing data from NCBI database with access number SRP076038 and computed the OTU tables with QIIME (Caporaso et al., 2010) software pipeline. Singleton OTUs (with singe read only) were discarded to remove their spurious effects.

### Sloan et al. (2006; 2006) Near Neutral Model

Sloan et al. (2006, 2007) derived a neutral model to explain the assembly mechanisms of prokaryotic communities. As a continuous version of Hubbell’s discrete neutral community model, Sloan’s model does not require observed species abundance distributions or patterns and can test very large prokaryotic communities. The model contains source and local communities, similar to “mainland” and “island” in the theory of island biogeography. We can first assume that the local community is saturated with N_*T*_ individuals. One individual dies or leaves the local community and is replaced by another individual immigrating from a source community with probability *m* or offspring of a random individual within local community with probability 1-*m*. Thus, the probability that the abundance of the *i-*th OTU increases by one individual, decreases by one individual, or shows no change can be given by:

(1) $$\text{Pr}\left(N_{i}+1/N_{i}\right)=\left(1-\frac{N_{i}}{N_{T}}\right)\left[mp_{i}+\left(1-m\right)\left(\frac{N_{i}}{N_{T}-1}\right)\right]$$

(2) $$\text{Pr}\left(N_{i}+1/N_{i}\right)=\frac{N_{i}}{N_{T}}\left[m\left(1-p_{i}\right)+\left(1-m\right)\left(\frac{N_{T}-N_{i}}{N_{T}-1}\right)\right]$$

$$\text{Pr}\left(N_{i}/N_{i}\right)=\frac{N_{i}}{N_{T}}\left[mp_{i}+\left(1-m\right)\left(\frac{N_{i}-1}{N_{T}-1}+\frac{N_{T}-N_{i}}{N_{T}}\right)\right]$$

(3) $$\left[m\left(1-p_{i}\right)+\left(1-m\right)\left(\frac{N_{T}-N_{i}-1}{N_{T}-1}\right)\right]$$

where *p*_*i*_ is the occurrence frequency of the *i*th OTU in the source community and *N*_*i*_ is the abundance of *i*th OTU in the local community. Let *x*_*i*_ = *N*_*i*_/*N*_*T*_ be the occurrence frequency of the *i*th OTU in the local community. The prediction abundance (*ϕ_*i*_*) of community is the beta distribution:

(4) $$\phi_{i}=cx_{i}^{N_{T}mp_{i}-1}{\left(1-x_{i}\right)}^{N_{T}m\left(1-p_{i}\right)-1}$$

$$\mathrm{where},c=\frac{\Gamma \left(N_{T}m\right)}{\Gamma \left[N_{T}m\left(1-p_{i}\right)\right]\Gamma \left(N_{T}mp_{i}\right)}.$$

From Sloan’s model, we can judge whether each species is neutral or not. According to Burns et al. (2016), the process for testing Sloan’s neutral model can be summarized as follows:

(1) Compute *p*_*i*_ and *x*_*i*_, fitting beta distribution and obtaining the estimation of *m*.
(2) Compute the theoretical occurrence frequency of species *i* across all local community samples with *m* and the beta distribution.
(3) Judge whether the observed *x*_*i*_ of species *i* falls within its 95% theoretical interval predicted from the neutral community model, and obtain a list of neutral, below neutral, and above neutral species.

We used Burns et al. (2016) scripts to implement the above procedures for fitting Sloan et al. (2006, 2007) near-neutral model.

### Multi-Site Neutral (MSN) Model

Harris et al. (2017) is an implementation of Hubbell (2001) unified neutral theory of biodiversity (UNTB) by approximating the multinomial (MN) species abundance distribution model with a hierarchical Dirichlet process (HDP). The derived algorithm can simultaneously estimate the migration rates among reasonable large number of sites, and therefore, we term the model as multi-site neutral model (MSN) or HDP approximated MSN model (HDP-MSN). With MSN modeling, the neutrality test can be performed at both local community and metacommunity level simultaneously. In other words, there are *P*-values for local community neutrality and metacommunity neutrality, respectively. For detailed computational procedures and software program of the MSN model, one may refer to Harris et al. (2017).

### Niche-Neutral Hybrid Model

Tang and Zhou (2013) proposed a hybrid niche-neutral model for multiple discrete communities developed by Volkov et al. (2007). Volkov et al. (2007) assumed that interspecies interactions in a steady-state community can be ignored and all species in the community become functionally equivalent. Here, based on the datasets of the healthy and breast tumor bacteria, we treated each sample as a niche occupied by a local microbial community and fit the neutral model for each local community. We used the *p*-value of the Chi-squared test to determine whether the metacommunity fit the NNH model. At the metacommunity level, if *p* > 0.05, the metacommunity satisfies the NNH and its assembly is co-driven by both niche and neutral processes, implying that the metacommunity itself does not satisfy the neutral theory, but within each niche, the local community is neutral; if *p* < 0.05, the metacommunity does not satisfy the NNH, implying that within each niche, the local community is not neutral either, and the metacommunity assembly is solely influenced by the niche process. The software program for implementing NNH model was reported by Tang and Zhou (2013).

### The Overall Modeling Design and Computational Implementations

Summarizing previous sub-sections for the breast tissue bacteria datasets, as well as the three metacommunity models, we still need to design computational implementations to apply the models to the datasets for achieving our objectives – assessing and interpreting the relative important of stochastic neutral drifts and deterministic selection (specifically tumor progression in this study). Table 1 below outlines our study design. An important step in our design was the adoption of random re-sampling (reallocations) of the samples for 1000 times from each of the three tissue categories (normal, benign, and malignant) or 100 times from each of the pair-wise two categories (to evaluate the directional changes). The re-sampling of 1000 (100) times was used build metacommunities, and therefore 1000 (100) metacommunity models (Sloan, MSN or NNH) were built for the whole datasets. The random re-sampling was used to raise the robustness of the modeling, in particular to compensate for the relative small sample sizes. Obviously, the tissue bacteria samples are much more difficult to obtain than those in non-invasive studies such as stool or oral bacteria sample collections.

**TABLE 1:** The study design for testing Sloan et al. (2006; 2006) near neutral, Harris et al. (2017) neutral and Tang and Zhou (2013) niche-neutral hybrid models for three types of breast tissue bacteria samples.

Scale	Sampling procedures	Models
Species Level	Select a group of community samples as the source community, another group as the destination (local) community, i.e., (*i*) Normal (source) to Benign (local) (*ii*) Normal (source) to Malignant (local) (*iii*) Benign (source) to Malignant (local)	Sloan’s near neutral model Sloan et al., 2006, 2007
Metacommunity Level	Randomly select one community sample from each of the three groups: 23 normal tissue samples, 12 benign tissue samples, and 33 malignant tissue samples. The sampling was performed with replacement, there were 23 × 12 × 33 = 9108 possible combinations (metacommunities). We randomly select 1000 out of the 9108 without replacement and fit the model to each of the 1000 metacommunities.	MSN Harris et al., 2017, NNH Tang and Zhou, 2013
	Randomly select one community sample from each of the three pairs of groups, i.e., {normal, benign} {normal, malignant} and {benign, malignant}, to form a metacommunity of two local communities. For each of the following three types of metacommunities, only 100 times of re-sampling were performed. That is, (*i*) From the combinations of the 23 normal tissue samples 12 benign samples, there were 23 × 12 = 144 possibly combinations (metacommunities), 100 metacommunities are randomly selected from the 144 possible combinations. Similarly, 100 metacommunities are selected from the following combinations: (*ii*) Normal (23) and Malignant (33), 23 × 33 = 759 (*iii*) Benign (12) and Malignant (33), 12 × 33 = 396	MSN Harris et al., 2017, NNH Tang and Zhou, 2013

## Results and Discussion

### Identifying Neutral, Negatively Selected (Below-Neutral) and Positively Selected (Above-Neutral) Species With Sloan et al. (2006; 2006) Near Neutral Model

**FIGURE 1:**
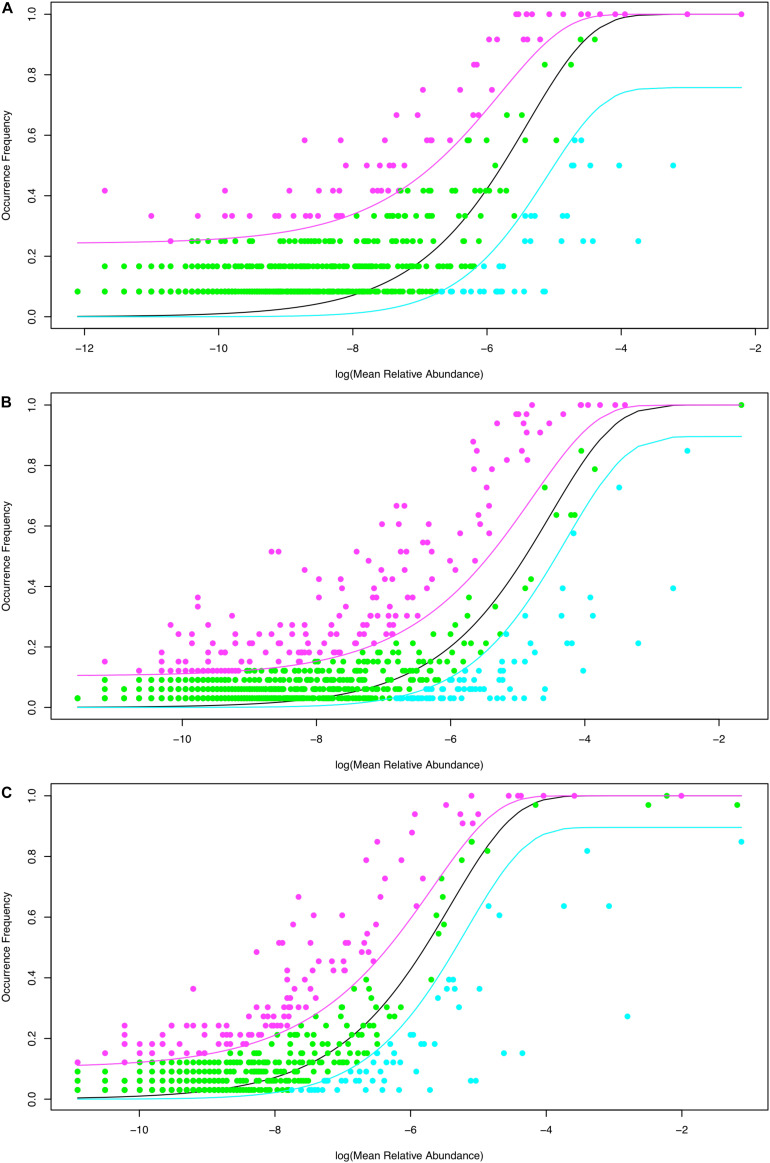
Fitting Sloan et al. (2007) near-neutral model to breast tissue bacteria datasets: The three lines (curves) are predicted with Sloan model. The black line is the predicted relative abundance, and the pink and cyan lines are the 95% confidence intervals. The pink points represent positively selected species with occurrence frequency greater than that of the neutral species (green points), and cyan points represent negatively selected species with occurrence frequency less than that of the neutral species (green points). **(A)** Treating the healthy tissue bacteria as source community and the benign tumor tissue bacteria as destination community. **(B)** Treating the healthy tissue bacteria as source community and the malignant tumor tissue bacteria as destination community. **(C)** Treating the benign tissue bacteria as source community and the malignant tumor tissue bacteria as destination community.

Using Sloan’s model, all species in a metacommunity can be classified into three groups: neutral, below neutral (negatively selected) and above neutral (positively selected). Table 2 listed the parameters of Sloan’ model. In Table 2, *N* represents the mean of bacterial individuals in source community, *m* represents immigrant probability from source community to destination community, *R*^2^ represents the goodness-of-fitting between observed and predicted frequencies with Sloan’s model. Table 2 also listed the percentage of neutral, below-neutral (negatively selected) and above-neutral (positively selected) species, respectively. As shown in Table 2, Supplementary Table 5A and Figure 1A, when we treated the normal tissue bacteria as source community and the benign tumor ones as destination community, 84.8% of the species belonged to neutral category, 5.6% were negatively selected species, and 9.6% were positively selected species. Given that the normal tissue bacteria were treated as source community, when we treated the malignant tumor ones as destination community, 76.5% of the species were driven by stochastic neutral forces, 8% were driven by negatively selected, and 15.5% were driven by positively selected (Table 2; Supplementary Table 5B; Figure 1B). We also tested Sloan’s model by treating the benign tumor tissue bacteria as the source community and the malignant tumor ones as the destination community, in which 75.2% of the species satisfy neutral theory, 8% belonged to negatively selected species, and 16.8% belonged to positively selected species (Table 2; Supplementary Table 5C; Figure 1C).

**TABLE 2:** Fitting of breast tissue bacteria datasets to Sloan et al.’s (2006; 2006) neutral model.

Source community	Destination community	*N*	*m*	R^2^	Total	Neutral (%)	Below neutral (%)	Above neutral (%)
Normal	Benign	15685.667	0.003	0.185	712	84.8	5.6	9.6
	Malignant	9120.485	0.002	0.347	1085	76.5	8.0	15.5
Benign	Malignant	9120.485	0.005	0.499	721	75.2	8.0	16.8

**FIGURE 2:**
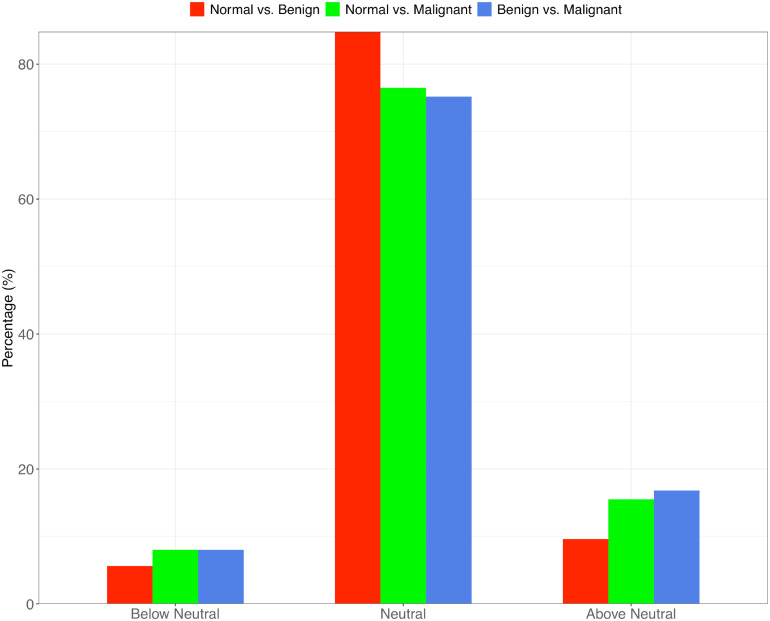
The percentage of neutral, below-neutral (negatively selected) and above-neutral (positively selected) species in the three types of metacommunity settings: “normal to benign,” “normal to malignant” and “benign to malignant.” The “benign to malignant” group shows the lowest neutral-species percentage but highest percentage of the above-neutral species.

From these results and the results of significance tests of difference, we found that the percentage of neutral species in tissue bacteria significantly decreased with the transformation of tumor from benign to malignant; meanwhile, the percentage of non-neutral species, especially positively selected species, significantly increased when malignant transformation occurred (Fisher test: *p-*values < 0.001; Figure 2). Specifically, in the breast tissue bacteria, approximately 10% of the species is positively selected by the progress of benign tumorigenesis, but the percentage of the species positively selected by malignant transformation is up to about 17%.

That decreasing in neutral species and increasing in non-neutral species revealed that selection forces became stronger with the development of breast cancer. All negatively (below-neutral) and positively (above-neutral) selected species were listed in Supplementary Table 5. We can infer that those negatively selected species were below the neutral predicted frequency because of unadaptable for benign or malignant transformation. Similarly, the positively selective species were better adaptation for tissues near breast cancer. So the selected non-neutral species may have stronger interaction with breast cancer than neutral species, which would be further lines of breast cancer research.

### Determining the Neutrality at Local Community and Metacommunity Levels With Harris et al. (2017) MSN (Multi-Site Neutral) Model

**FIGURE 3:**
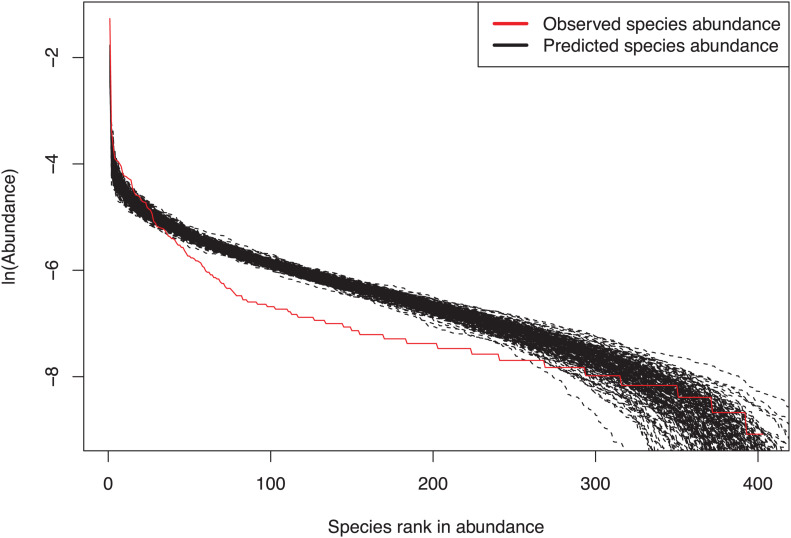
An example illustrating the fitting of the MSN (multi-site neutral) model with the breast tissue bacteria: three samples, one from each of the three categories of samples (normal, benign and malignant), constitute a multi-site metacommunity.

We fitted the Harris et al.’s MSN model to four multi-site metacommunity settings (groups). The first group consisted of three bacteria from normal, benign tumor and malignant tumor tissues, respectively (Group name: “normal & benign & malignant”). The full results of fitting the MSN model to this group were listed in Supplementary Table 1. Each of other three groups consisted of two bacteria from two types of breast tissues. The group names of these three meta-communities were “normal & benign,” “normal & malignant” and “benign & malignant,” The full results of fitting to these three groups were listed in Supplementary Table 2. Table 3 listed the mean parameters and corresponding standard errors of MSN models fitted to the breast bacteria, which were summarized from Supplementary Tables 1, 2. Figure 3 shows an example of fitting the MSN model with the dataset of “normal & benign & malignant” group.

**TABLE 3:** The summary results of fitting Harris *et al’s* (2017) HDP-MSN (hierarchical *Dirichlet* process, multi-site neutral) model to the breast bacteria, excerpted from Supplementary Tables 1, 2 in the OSI where the full fitting results from 1000 or 100 times of re-sampling were exhibited*.

Group		*L*_*O*_	*θ*	*M-value*	Meta-community				Local community			
					*L*_*M*_	*N*_*M*_	*N*	*p*_*M*_	*L*_*L*_	*N*_*L*_	*N*	*p*_*L*_
Normal & Benign & Malignant	Mean	−5597.422	771.918	67.704	−5130.309	149	2500	0.060	−5245.885	183	2500	0.073
	Std. Err.	68.235	11.424	0.824	58.384	9.012	0	0.004	61.189	6	0	0.002
Normal & Benign	Mean	−3833.236	637.265	73.711	−3431.210	90	2500	0.036	−3545.737	146	2500	0.058
	Std. Err.	164.272	30.268	3.555	140.811	17.8	0	0.007	147.599	13.8	0	0.006
Normal & Malignant	Mean	−3500.268	544.794	75.589	−3118.059	100.9	2500	0.040	−3220.030	122.2	2500	0.049
	Std. Err.	160.803	28.785	3.710	137.605	19.9	0	0.008	144.640	13.9	0	0.006
Benign & Malignant	Mean	−2825.108	501.805	54.321	−2573.222	163.1	2500	0.065	−2651.723	270.9	2500	0.108
	Std. Err.	73.616	21.344	1.690	65.218	23.2	0	0.009	68.602	21.9	0	0.009

*N = 2500 is number of Gibb samples selected from 25,000 simulated communities (i.e., every tenth iteration of last 25,000 Gibbs samples), chosen to compute pseudo p-value for conducting the neutrality test. L_0_ is actual log-likelihood, computed from median of 25,000 simulations and compared with log-likelihood of each simulated community. θ is median of biodiversity parameters computed from 25,000 simulations. m is migration probability. M is average median of migration rates of local communities in each metacommunity (i.e., average median of individuals migrated per generation), computed from 25,000 simulations. L_M_ is median of log-likelihoods of simulated neutral metacommunity samples. N_M_ is number of simulated neutral metacommunity samples with likelihoods not exceeding the actual likelihood, L ≤ L_0_. P_M_ = N_M_ / N is pseudo p-value for testing neutrality at metacommunity level; if p_M_ > 0.05, metacommunity satisfies MSN model. L_L_ is median of log-likelihoods of simulated local community samples, and N_L_ is number of simulated local community samples with likelihoods not exceeding L_0_. P_L_ = N_L_ / N is pseudo p-value for testing neutrality at local community level; if p_L_ > 0.05, local community satisfies neutral model.). The P_M_-values exhibited here are adjusted as (P_M_ = 1-P_MS_), where P_MS_ is output from Harris et al. (2017) computational program. Similarly, the P_L_-values are adjusted as (P_L_ = 1-P_LS_), where P_LS_ is output from their computational program.

**FIGURE 4:**
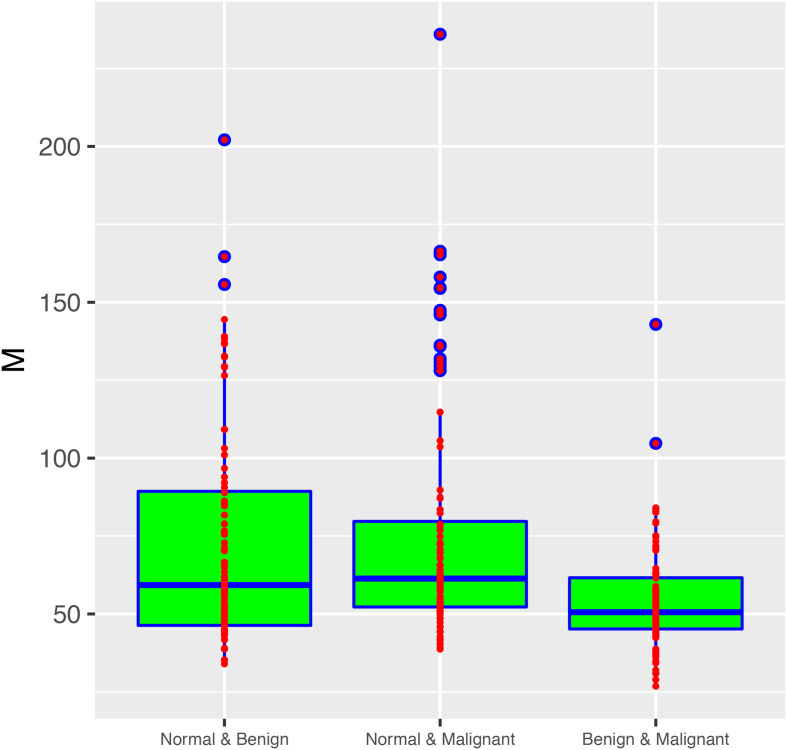
The box plot showing the fundamental dispersal number (*M*) in three metacommunity settings (groups): “normal & benign,” “normal & malignant” and “benign & malignant.” The rightmost group (benign & malignant) exhibited significantly smaller *M*-value than the two other groups (Wilcoxon test: *P*-value < 0.001). There was no significant difference between the other two groups in their *M*-values (*P*-value = 0.348). Three standard summary numbers (statistics) of the parameter (*M*), including the first quartile (lower edge of the rectangle), median (the inside segment), third quartile (upper edge of the rectangle) were displayed, respectively. The “whiskers” above and below the box (rectangle) show the location of the minimum and maximum. The inter-quartile range (IQR) (showing the range of variation) is displayed by the height of the box; and the median shows the typical value. Outliers (<3×IQR or >3×IQR) are displayed outside the box.

From these results, we found that 33.6% of all 1000 sampling passed the neutrality test with the MSN model. It suggested that, at community level, the community assembly and diversity maintenance of breast tissue bacteria were influenced by stochastic neutral forces, including dispersal and drift. We therefore postulate that the stochastic dispersal and drift is likely to play an important role in the tumor development. Table 3 also lists the average migration rates (*M*-values) between two types of breast tissue bacteria. The microbial migration rates between normal and two types of tumor tissue were similar without significant difference (Wilcoxon test: *p*-value = 0.348 > 0.05), and the average *M* of them was 74.65. The migration rate between bacteria of two tumor tissues was 54.321, and was significantly smaller than *M*-values of “normal & benign” and “normal & malignant” (Wilcoxon test: *p*-value < 0.001, Figure 4). The lower *M* value represents more similar structure and components of microbial communities between benign and malignant groups.

Figure 3 shows fitting of MSN to #998 sample by plotting predicted and observed species abundance rank distributions.

### Determining the Balance Between Niche Selection and Neutral Drift With Tang and Zhou (2013) NNH (Niche-Neutral Hybrid) Model

Similarly to the design of fitting the MSN model, we fitted the NNH model to four meta-community groups: “normal & benign & malignant,” “normal & benign,” “normal & malignant,” and “benign & malignant.” The full results of fitting the NNH model to group of “normal & benign & malignant” were listed in Supplementary Table 3, and to other three groups were listed in Supplementary Table 4. Table 4 listed the average NNH parameters and corresponding standard errors, which are summarized from Supplementary Tables 3, 4.

**TABLE 4:** The summary results of fitting Tang and Zhou (2013) NNH (niche-neutral hybrid) model to the breast bacteria, excerpted from Supplementary Tables 3, 4 in the OSI where the full fitting results from 1000 or 100 times of re-sampling were exhibited*.

Group		*J*	*S*	*θ*	*m*	*x*	*γ*	*R* ^2^	*χ^2^*	*p*-value	N_pass_	%(pass)
Normal & benign	Mean	17022.2	267.730	79.143	0.000	0.840	0.966	0.423	236.880	<0.001	0	0
	Std. Err.	1201.096	10.983	2.980	0.000	0.006	0.048	0.010	17.418	0.000	0	0
Normal & malignant	Mean	11605.9	252.500	75.978	0.000	0.838	0.980	0.389	215.120	<0.001	0	0
	Std. Err.	935.399	15.140	3.733	0.000	0.006	0.047	0.010	17.776	0.000	0	0
Benign & malignant	Mean	12765.1	207.165	70.491	0.000	0.836	0.935	0.477	174.578	<0.001	0	0
	Std. Err.	739.849	4.859	1.984	0.000	0.005	0.039	0.009	7.280	0.000	0	0
Three groups	Mean	13144.6	243.646	74.864	0.000	0.834	0.992	0.457	316.3	<0.001	0	0
	Std. Err.	216.797	2.627	0.763	0.000	0.001	0.012	0.003	5.713	0.000	0	0

*J, average number of individuals per niche (local community) in each metacommunity; S, average species number per niche (local community) in each metacommunity; θ, average fundamental biodiversity parameter per niche (local community) in each metacommunity; m, average of migration coefficients, x, average of birth to death ratio; γ, average of migration rate; R^2^, goodness-of-fit index; χ^2^, χ^2^-value of chi-squared test for observed value against predicted value; p-value for χ^2^-test; when p > 0.05, metacommunity satisfies NNH model. Last two columns are number and percentage of local communities (niches) that passed local neutrality test.

From these results, we found that there was no tested dataset passing the test with the NNH model (*p*-values < 0.001). It further verified the previous finding, i.e., stochastic forces or neutral drift plays an important role in shaping the structure and diversity of breast tissue bacteria, while niche-differentiations or deterministic selection forces from tumor formation and malignant transformation plays little role.

### The Dispersal (Migration) Patterns of Breast Tissue Bacteria

In previous sections, based on Sloan near neural model (Table 2), we have demonstrated that while neutral species constitute, on average, approximately 85% species in the breast tissue bacteria, the selection effect from tumor progression does lead to certain percentage of above-neutral or positively selected species. The percentage of positively selected species is about 10% on average, but could be up to about 17% in the progression to malignant tumor. The MSN/NNH models, nevertheless, showed that the selection is not sufficiently strong to lead to community/meta-community level dominance of stochastic neutral forces. In other words, whereas the breast tissue bacteria are driven predominantly by stochastic neutral forces, at the species level, up to 17% approximately of species can be positively selected by tumor progression.

With MSN model, we can further infer the migration rate between breast tissue bacteria. Figure 4 shows the fundamental dispersal number or the migration rate (*M*) of pair-wise microbial migration between two types of breast tissue bacteria (normal tissue and benign tumor; normal and benign tumor, benign and malignant tumor). Average *M*-values were previously displayed in Table 3 and detailed *M*-values were displayed in Supplementary Tables 1, 2. The non-parametric Wilcoxon tests revealed no significant difference in the *M*-value between “normal & benign” and “normal & malignant” (*p* = 0.348), but the difference in *M* was significant between “benign to malignant” and the two other groups mentioned above (*P*-value < 0.001). This may indicate a significant difference between the benign and malignant tumor tissues in their microbial dispersal.

## Conclusion and Limitations

The three neutral theoretic models (MSN, NNH and Sloan near-neutral model) we used in this study each focused on different aspects of community assembly. MSN model performed neutrality test at local and metacommunity levels. NNH model tested niche-neutral hybrid or continuum at metacommunity level. The Sloan model focused on neutral vs. selection from species-level perspective and split species into neutral, below neutral and above neutral groups. Their results complemented with each other. The results from MSN and NNH suggested that stochastic neutral drifts were in effects in approximately 1/3 of tested groups involving tumor development. The species-level results with Sloan model suggested that the proportion of neutral species exceeded 75%, and proportions of selected species were under 25 with positively selected species being moderately more than negatively selected species. In conclusion, we believe that both stochastic drifts and deterministic selections are important in shaping the structure and dynamics of breast tissue bacteria, including in influencing tumor development.

The limitations of this study include relatively small sample sizes. Another limitation was that the tissue samples were not explicitly matched to individual subjects, possibly due to privacy concerns. For the first issue, we are collecting more datasets from larger cohort studies and hope to further verify our findings reported in this preliminary report. For the second issue, we have adopted 1000 times of re-sampling of the samples from their possible permutations. Since the 1000 times of re-sampling exceed the number of all possible permutations of the samples, the issue becomes largely irrelevant when we built 1000 sets of MSN/NNH models (one for each re-sampling) and took the average model parameters for inferences.

## Data Availability Statement

All datasets analyzed in this study are available in NCBI database with accession number SRP076038 deposited by Urbaniak et al. (2016).

## Author Contributions

LL: software, data curation, formal analysis, and writing – original draft. PN: conceptualization and reviewing the manuscript. ZM: conceptualization, methodology, and writing – reviewing, and editing. All authors contributed to the article and approved the submitted version.

## Conflict of Interest

The authors declare that the research was conducted in the absence of any commercial or financial relationships that could be construed as a potential conflict of interest.
